# In vivo competition assays between Vip3 proteins confirm the occurrence of shared binding sites in *Spodoptera littoralis*

**DOI:** 10.1038/s41598-022-08633-y

**Published:** 2022-03-17

**Authors:** María Lázaro-Berenguer, Yudong Quan, Patricia Hernández-Martínez, Juan Ferré

**Affiliations:** Institute of Biotechnology and Biomedicine (BIOTECMED), Department of Genetics, Universitat de València, 46100 Burjassot, Spain

**Keywords:** Biochemistry, Proteins, Biotechnology, Plant biotechnology, Microbiology, Applied microbiology

## Abstract

Due to their different specificity, the use of Vip3 proteins from *Bacillus thuringiensis* in combination with the conventionally used Cry proteins in crop protection is being essential to counteract the appearance of insect resistance. Therefore, understanding the mode of action of Vip3 proteins is crucial for their better application, with special interest on the binding to membrane receptors as the main step for specificity. Derived from in vitro heterologous competition binding assays using ^125^I-Vip3A and other Vip3 proteins as competitors, it has been shown that Vip3 proteins share receptors in *Spodoptera frugiperda* and *Spodoptera exigua* brush border membrane vesicles (BBMV). In this study, using ^125^I-Vip3Aa, we have first extended the in vitro competition binding site model of Vip3 proteins to *Spodoptera littoralis*. With the aim to understand the relevance (in terms of toxicity) of the binding to the midgut sites observed in vitro on the insecticidal activity of these proteins, we have performed in vivo competition assays with *S. littoralis* larvae, using disabled mutant (non-toxic) Vip3 proteins as competitors for blocking the toxicity of Vip3Aa and Vip3Af. The results of the in vivo competition assays confirm the occurrence of shared binding sites among Vip3 proteins and help understand the functional role of the shared binding sites as revealed in vitro.

## Introduction

Genetically modified crops expressing Cry insecticidal protein genes from *Bacillus thuringiensis* (Bt crops), have been used since the mid 90’s to control insect pests. However, their extensive use has led to the phenomenon of field-evolved resistance to Cry proteins, for some lepidopteran and coleopteran pests^1–3^. Pyramided Bt crops (which combine different insecticidal proteins with different specificity and mode of action) have been proposed as an interesting tool to delay or avoid the appearance of insect resistance^4,5^. In this context, Vegetative Insecticidal Proteins (Vip3), known as a second generation of Bt proteins, are gaining importance in crop protection strategies. Most studies have dealt with Vip3Aa proteins, which are already expressed in commercial Bt crops, are active against lepidopteran species that have already developed resistance against the Cry proteins, and do not share binding sites with them^6–10^.

Vip3 proteins have a tetrameric structure, with each monomer containing five structural domains^11–13^. The N-terminal (Nt) domains I–III are highly conserved and play a crucial role in maintaining the tetramer^12,14^. Domain I has been proposed to form, upon activation, a coiled-coil that inserts into the epithelial membrane^11,12^, and domain III has recently been proposed to be responsible of binding to the midgut receptors^6,15^. The C-terminal (Ct) domains IV–V are highly variable sequence and are not essential for maintaining the tetrameric structure, though they are critical for the insecticidal activity in vivo^14,16^. These domains are carbohydrate binding modules (CBM) whose function remains still unknown^11–13^.

In vitro binding assays to midgut binding sites have been used to establish binding site models which are useful to predict the appearance of cross-resistance and select the best protein combinations for pyramided Bt crops^8,10,17^. More recently, in vivo competition assays have been proposed as a novel tool to complement the in vitro binding models and obtain information about protein interactions in vivo^18–21^. Studies based on in vitro binding of ^125^I-Vip3A to brush border membrane vesicles (BBMV) using heterologous Vip3 proteins as competitors led to propose a binding site model in which Vip3 proteins share binding sites in *Spodoptera frugiperda* and *Spodoptera exigua*^6,7^. In the present study, using ^125^I-Vip3Aa, we have extended the in vitro binding site model of Vip3 proteins to another Spodoptera species, *Spodoptera littoralis*, with the aim to understand the relevance of binding to the midgut binding sites observed in vitro on the insecticidal activity of these proteins. For this purpose, we have performed in vivo competition assays with *S. littoralis* larvae, using Vip3 Disabled Insecticidal Proteins (DIP) carrying mutations in domain I as competitors for blocking the toxicity of Vip3Aa and Vip3Af. DIP mutants are engineered in such a way that they lack insecticidal activity without losing the capacity of binding to membrane receptors with the aim to see if they compete with wild type (WT) proteins in vivo for functional binding sites, as detected in toxicity assays^18,21^. The results of our in vivo competition assays confirm the occurrence of functional shared binding sites for Vip3 proteins and confirm the binding site model proposed by in vitro binding using ^125^I-labeled Vip3 proteins*.*

## Results

### Toxicity of Vip3 WT and domain I mutant proteins

Surface contamination bioassays were performed to determine the toxicity of Vip3 WT proteins and that of the disabled proteins against *S. littoralis* neonate larvae (Table 1). LC_50_ values (concentration killing 50% of the larvae) of the WT proteins served to establish the doses to perform the in vivo competition assays. The mutants carrying mutations in domain I, used as competitors for the in vivo competition assays, lacked detectable insecticidal activity (Table 1)*.* The results indicate that Vip3Aa and Vip3Af proteins are both highly toxic for *S. littoralis*, whereas Vip3Ca was very little toxic though we could observe a strong growth inhibition as already reported by Palma et al.^22^.

**Table 1:** Toxicity of Vip3 proteins against *S. littoralis* neonate larvae.

Protein	LC_50_ (FL 95%) (ng/cm^2^)^a^	Slope ± SEM	χ^2^
Vip3Aa WT	13.9 (5.8–41.7)	2.4 ± 0.6	5.1
Vip3Aa DIP1 (S164C L166C)	> 13,000	–	–
Vip3Aa DIP2 (E168A)	> 30,000	–	–
Vip3Af WT	28.8 (22.7–39.3)	2.3 ± 0.2	3.0
Vip3Af DIP (E168A)	> 30,000	–	–
Vip3Ca WT	> 1000	–	–
Vip3Ca DIP (S164C L166C)	> 30,000	–	–

^a^The LC_50_ and the 95% fiducial limits (FL 95%) after 10 days. At least three biological replicates were performed for each protein except Vip3Ca WT which was estimated from a single assay.

### ^125^I-Vip3Aa in vitro binding assays

**Figure 1:**
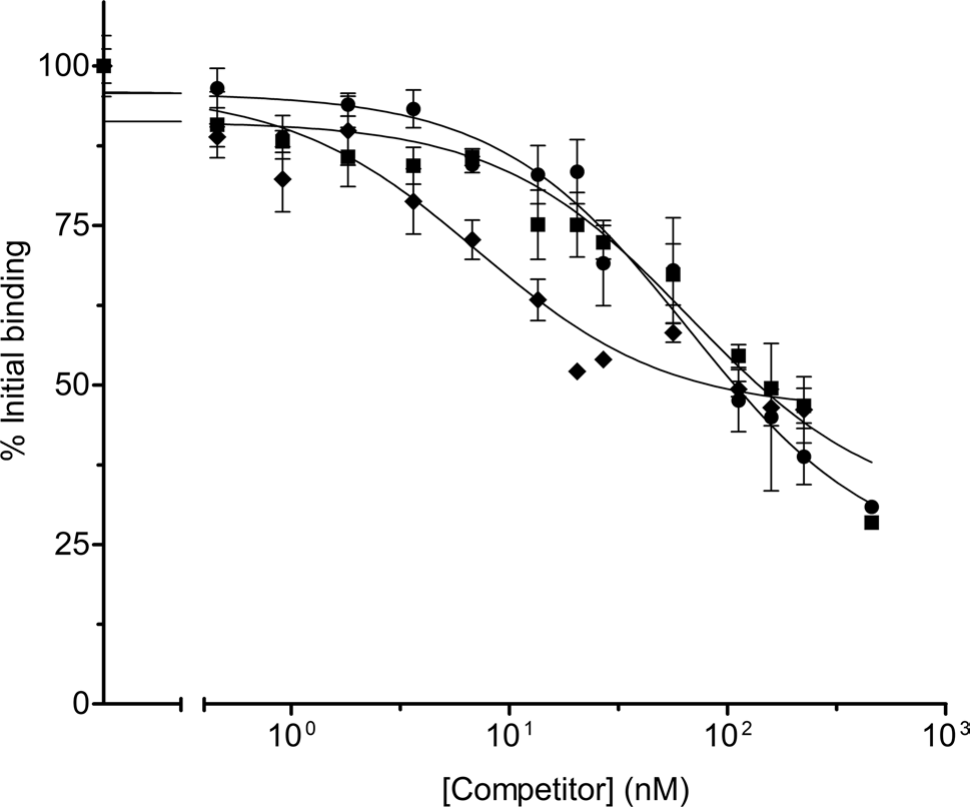
In vitro binding competition assays among WT Vip3 proteins. Labelled ^125^I-Vip3Aa was used with a fixed concentration of BBMV (0.05 mg/ml) and increasing concentrations of unlabelled Vip3Aa (circles) and the heterologous proteins Vip3Af (squares) and Vip3Ca (diamonds) as competitors. Each data point represents the mean of at least three replicates (± SEM).

Specific binding of ^125^I-Vip3Aa to *S. littoralis* BBMV was shown by the competition by unlabeled Vip3Aa (Fig. 1, circles; Supplementary Fig. S1). The unlabeled protein displaced the labelled protein up to almost 30%, indicating that this was the percentage of non-specific binding in our experimental conditions. The analysis of the competition curve provided an equilibrium dissociation constant (*K*_*d*_) of 51.0 ± 8.3 nM, with a concentration of binding sites (*R*_*t*_) of 170 ± 32 pmol/mg of BBMV protein (Supplementary Table S1). Heterologous competitors (Vip3Af and Vip3Ca) were used to see whether they competed with ^125^I-Vip3Aa binding, an indication that they would share binding sites with this protein. The results showed that Vip3Af was able to displace all ^125^I-Vip3Aa specific binding (Fig. 1, squares) in a similar way as Vip3Aa does. The analysis of the heterologous curve using Vip3Af WT as competitor yielded an estimated *K*_*d*_ of 130 ± 39 nM and an *R*_*t*_ of 492 ± 202 pmol/mg of BBMV protein (Supplementary Table S1). However, though Vip3Ca could compete ^125^I-Vip3Aa binding, it could not do it completely (Fig. 1, diamonds); the competition curve reaches a plateau at 50% binding which indicates that Vip3Aa had more than one population of binding sites and that not all are shared with Vip3Ca. Accordingly, the analysis of the heterologous curve using Vip3Ca WT as competitor yielded an estimated *K*_*d*_ of 6.6 ± 1.3 nM and an *R*_*t*_ of 18.3 ± 3.4 pmol/mg of BBMV protein (Supplementary Table S1), indicating a high affinity for the receptors shared with Vip3A proteins. However, according to the low *R*_t_ value, Vip3Ca protein only shares with Vip3A proteins a small number of binding sites.

### Effect of domain I mutations on in vitro binding

**Figure 2:**
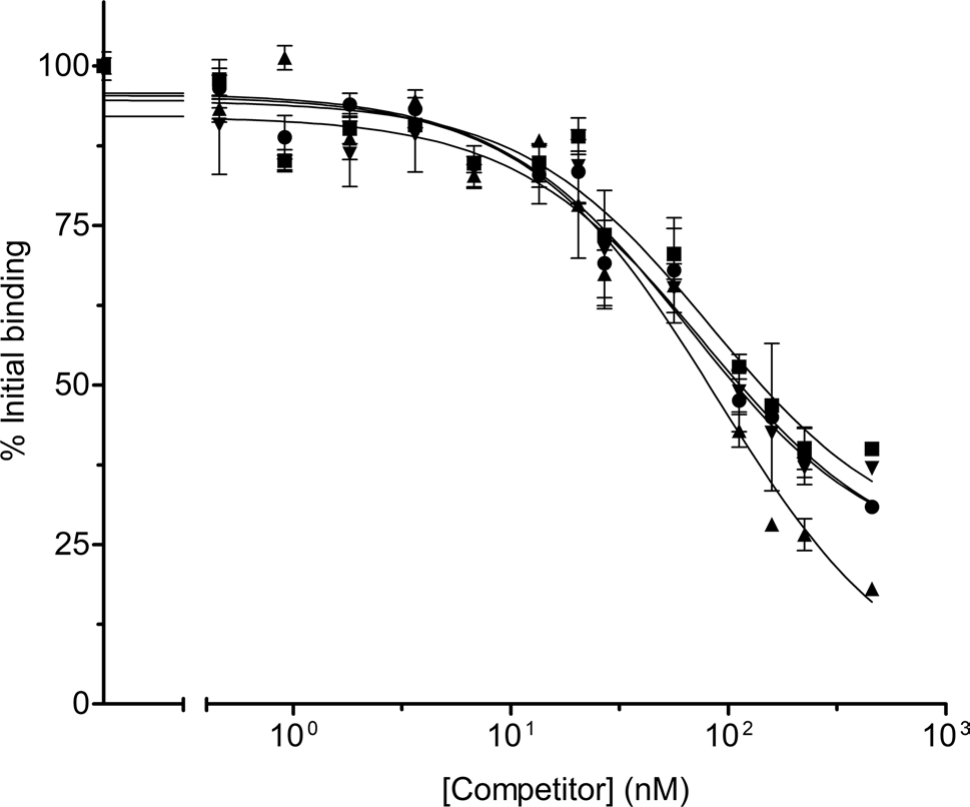
In vitro binding competition assays of domain I Vip3 mutants. Labelled ^125^I-Vip3Aa was used with a fixed concentration of BBMV (0.05 mg/ml) and increasing concentrations of unlabelled Vip3A domain I mutants: Vip3Aa DIP1 (triangles), Vip3Aa DIP2 (inverted triangles, overlapped with that of circles), and Vip3Af DIP (squares). The homologous competition with unlabelled Vip3Aa WT (circles, overlapped with that of triangles) is included as a control. Each data point represents the mean of at least three replicates (± SEM).

The ability of domain I mutant proteins to bind to BBMV has been shown by using ^125^I-Vip3Aa and the unlabeled mutant proteins as competitors. The results showed that all the tested mutants were able to compete with the labelled protein similarly to the unlabelled Vip3Aa (Fig. 2), indicating that they maintained the ability to bind to the binding sites of Vip3Aa despite having lost their insecticidal activity. The estimation of the binding parameters (*K*_*d*_ and *R*_*t*_) from the heterologous competition with ^125^I-Vip3Aa indicated that the mutations did not significantly affect the binding properties compared to their respective WT proteins (Supplementary Table S1**)**.

### Vip3Aa in vivo competition assays

**Figure 3:**
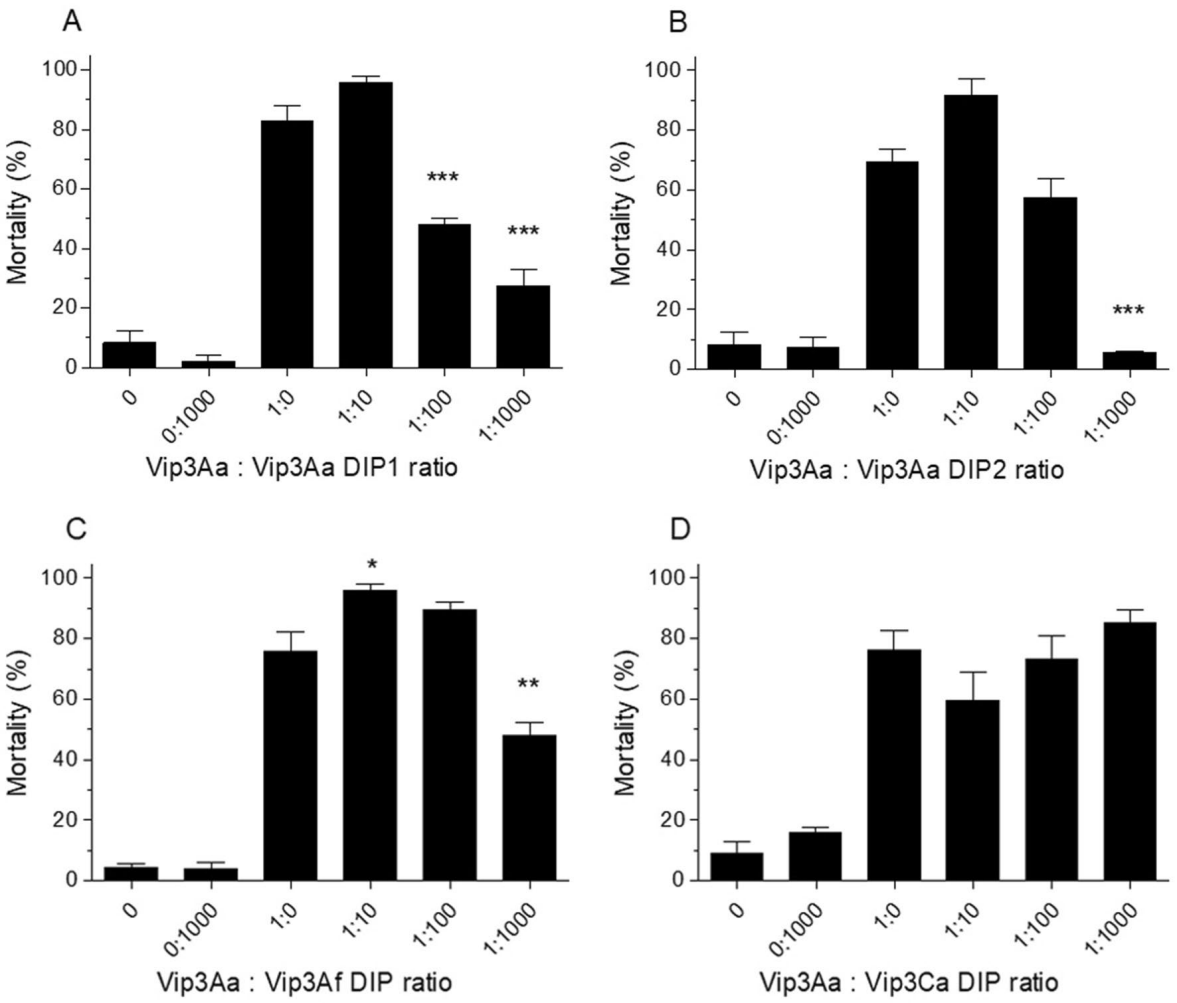
Vip3Aa in vivo competition assays against domain I mutants. Vip3Aa WT protein was used at a constant concentration (13 ng/cm^2^) with increasing ratios of homologous Vip3Aa mutant competitors (**A**) Vip3Aa DIP1 and (**B**) Vip3Aa DIP2, and heterologous competitors (**C**) Vip3Af DIP and (**D**) Vip3Ca DIP. The mean percent mortality (± SEM) of at least three replicates is represented for each protein ratio. The two first columns of each graph correspond to the negative controls 0 (just buffer) and 0:1000 (no WT but highest amount of competitor used in the assay, 13 µg/cm^2^). The rest of the columns represent the different protein:competitor ratios used in the assays. The asterisks over the bars indicate level of significance (*P < 0.05, **P < 0.01 and ***P value < 0.001) compared to the ratio 1:0 in a One way Anova with Bonferroni post hoc test.

In vivo competition assays were performed to test whether the in vitro competition binding model drawn using Vip3 heterologous competitors actually reflected competition for functional binding sites. At ratios 1:100 or higher of WT:mutant protein, both Vip3Aa domain I mutants (Vip3Aa DIP1 and Vip3Aa DIP2) were able to block the toxicity of Vip3Aa WT (Fig. 3A,B). Therefore, both disabled Vip3Aa mutants compete with the WT protein presumably by binding to the same binding sites, preventing Vip3Aa WT to exert its toxic action.

The same type of approach was used with heterologous Vip3 competitors (Vip3Af DIP and Vip3Ca DIP). The Vip3Af mutant protein was able to block Vip3Aa WT toxicity in vivo at the highest ratio tested (1:1000), whereas the Vip3Ca mutant was not able to block the toxicity of Vip3Aa WT (Fig. 3C,D).

### Vip3Af in vivo competition assays

**Figure 4:**
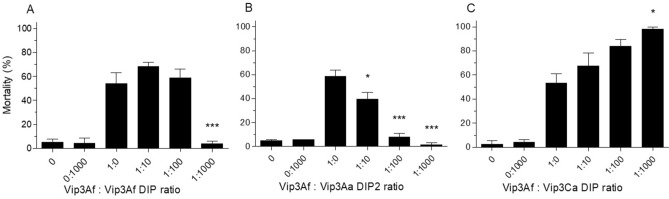
Vip3Af in vivo competition assays against domain I mutants. Vip3Af WT protein was used at a constant concentration (30 ng/cm^2^) with increasing ratios of homologous mutant competitor (**A**) Vip3Af DIP, heterologous mutant competitors (**B**) Vip3Aa DIP2 and (**C**) Vip3Ca DIP. The mean percent mortality (± SEM) of at least three replicates is represented for each protein ratio. The two first columns correspond to the negative controls 0 (just buffer) and 0:1000 (no WT but highest amount of competitor used in the assay, 30 µg/cm^2^). The rest of the columns represent the different protein:competitor ratios used in the assay. The asterisks over the bars indicate level of significance (*P < 0.05, and ***P < 0.001) compared to the ratio 1:0 in a One way Anova with Bonferroni post hoc test.

In vivo competition assays were also performed using the Vip3Af protein as the WT protein. In the homologous competition assay using the Vip3Af DIP mutant, the competitor blocked the toxicity of Vip3Af WT at a 1:1000 ratio (Fig. 4A). In agreement with the results obtained in the in vivo competition assays with Vip3Aa WT, Vip3Aa DIP2 blocked the toxicity of Vip3Af WT (Fig. 4B), whereas Vip3Ca DIP did not block it even at the highest ratio tested (Fig. 4C). Interestingly, Vip3Aa DIP2 was very efficient significantly blocking the toxicity of Vip3Af WT starting at a ratio as low as 1:10.

## Discussion

Understanding Vip3 proteins mode of action is essential for guiding a better application of these insecticidal proteins in pest management and crop protection strategies. Special emphasis must be placed on their binding properties, which drive their insect specificity and help designing the best protein combinations for pyramided Bt crops. In vitro binding assays with radiolabelled proteins has proven to be a useful method for studying protein binding to insect midgut receptors^8,10,17^. Here, by using ^125^I-Vip3Aa and *S. littoralis* BBMV in in vitro binding assays we have shown that Vip3Aa and Vip3Af proteins share all the binding sites whereas Vip3Ca only competes partially for Vip3A binding sites. This binding model is in agreement with the one proposed by Quan et al*.*^6^ for *S. exigua* and *S. frugiperda* using ^125^I-Vip3Af. The partial competition of Vip3Ca for Vip3A binding sites was not detected in a previous study with *S. frugiperda* BBMV^23^. In that study the binding conditions were different from ours, such as the lower purity of the proteins used as competitors and the presence of NaCl in the binding buffer, which has been shown to interfere with Vip3Af binding^6^.

Disabled insecticidal proteins have been proposed as a novel tool for studying the occurrence of shared receptors between different proteins in vivo^18,21^. The disabled mutants are proteins which have been engineered to carry mutations that eliminate their insecticidal activity without affecting their ability to bind to membrane receptors^18,21^. We have now applied this approach to Vip3 proteins in *S. littoralis* with the aim to test the relevance of in vitro derived binding site models to predict the interaction of these proteins in vivo. Vip3 disabled proteins where generated by the introduction of mutations in domain I and they were used as competitors in in vivo competition assays, which consist on giving a constant dose of a toxic protein and increasing concentrations of the disabled competitor. If the toxicity is blocked by an excess of competitor, it can be assumed that the proteins share functional receptors, and thus, the binding of the non-toxic protein prevents the binding and the subsequent toxicity of the wild type protein^18–21^. However, in this type of approach, a dominant-negative phenotype can be formed if, at very low concentrations of nontoxic competitor, an important inhibition of toxicity is observed, indicating that the competitor is sequestering the WT protein by forming hetero-oligomers^24–26^. This is not the case here, since Vip3 proteins spontaneously form very stable homotetramers and because the inhibition of toxicity at very low concentrations of competitors is not observed. Our results show that, as expected, the Vip3 disabled proteins carrying point mutations in domain I were all able to bind specifically to *S. littoralis* BBMV in the in vitro binding competition assays, but none of them was toxic against *S. littoralis* larvae, confirming the essential role that domain I plays on the toxicity^16,21^.

From the in vivo competition assays we can draw some conclusions that allow us to better understand the behaviour of Vip3 proteins in vivo. Disabled Vip3Aa and Vip3Af proteins blocked the toxicity of their respective homologous WT proteins, though they did it at different ratios. Whereas disabled Vip3Aa proteins blocked Vip3Aa WT at a 1:100 ratio, a 1:1000 ratio was needed for disabled Vip3Af to block Vip3Af toxicity. In the heterologous competition assays, whereas disabled Vip3Aa DIP2 blocked Vip3Af WT at a ratio 1:10, disabled Vip3Af DIP blocked Vip3Aa WT only at a ratio as high as 1:1000. The fact that Vip3Aa and Vip3Af disabled proteins blocked each other’s WT proteins confirms the occurrence of shared binding sites in vivo, in agreement with the in vitro binding assays. Furthermore, the different ratios WT:disabled required to block toxicity reveal differences in the efficiency between Vip3Aa and Vip3Af proteins, being disabled Vip3Af less efficient than disabled Vip3Aa in competing for the wild type proteins. These results are in agreement with the differences in affinity of these two proteins for the binding sites observed in vitro. Vip3Af has a higher *K*_*d*_ than Vip3Aa, indicating lower affinity for the binding sites, in agreement with the lower efficiency in blocking the toxicity in the in vivo competition assays.

Despite Vip3Ca being able to compete in vitro for part of the binding sites of Vip3Aa, the disabled Vip3Ca did not block the toxicity of either of these two proteins even at the highest ratio tested in in vivo competition (Figs. 3D and 4C). The partial competition of Vip3Ca in vitro for Vip3A binding sites implies that the latter have distinct types of binding sites, in agreement with recent reports identifying different receptors for Vip3A proteins^27–30^. Therefore, one explanation for the result obtained in vivo is that Vip3Ca cannot block all functional binding sites of Vip3A proteins and, thus, the non-shared binding sites are sufficient for Vip3A proteins to exert their toxic effect. Alternatively, the Vip3A binding sites shared with Vip3Ca may be those of less relevance for Vip3A toxicity or those occurring in less abundance compared to other Vip3A sites. Since Vip3Ca was not labelled (for the in vitro binding assays) or used as the WT protein in the in vivo competition assays, we cannot discard that it could have additional binding sites not shared with Vip3A proteins.

The existence of redundant binding sites for Vip3A proteins could also help explain the fact that resistance to Vip3Aa has never been associated with a lack of in vitro binding to BBMV in any of the lepidopteran Vip3Aa-resistant populations studied^31–33^.

An unexpected result observed in our in vivo competition assays using disabled Vip3A proteins as competitors is the increase of toxicity of the WT protein when mixed with the disabled protein at low ratios (Figs. 3C and 4C). One possible explanation for this effect is that the excess of disabled protein would block non-specific binding sites that otherwise would trap some of the WT molecules. Also, the excess of disabled proteins could have a “protective” effect to the WT protein in the lumen by reducing the ratio proteases:Vip3A molecules. This slight enhancement of the mortality disappears when the ratio of disabled protein increases due to the greater contribution of the binding site blocking effect, except when using Vip3Ca DIP as competitor since this protein is unable to block the WT binding sites.

**Figure 5:**
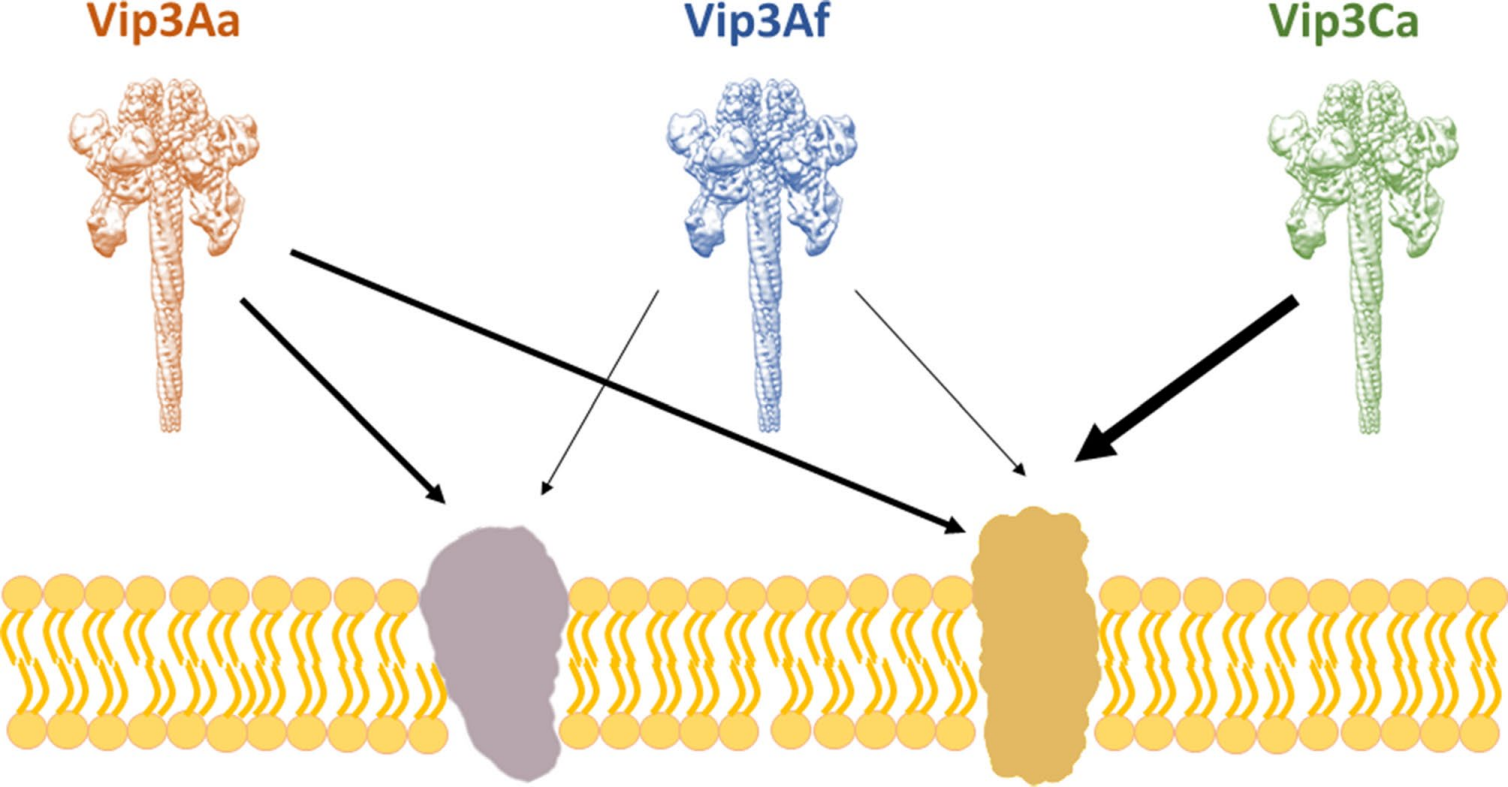
Vip3 binding site model for *S. littoralis*. Proposed binding site model based on in vitro binding of ^125^I-Vip3Aa and in vivo competition assays using Vip3A WT proteins. The width of the arrows represents differences in the binding affinity.

As a summary, the in vivo competition assays are a good complement to the in vitro competition binding assays. In this study, in addition to propose a binding site model for *S. littoralis* based on in vitro binding competition using radiolabelled proteins, the in vivo competition assays confirm the occurrence of functional shared binding sites for Vip3 proteins. The results point out to the occurrence of multiple binding sites, which might have different relevance in the toxic process or different abundance in the midgut membrane (Fig. 5). The occurrence of redundant receptors may have the effect of masking the importance of the ones most involved in the toxicity by providing an “average picture” of the overall binding to the membrane sites. Here, we propose the model shown in the Fig. 5 as the most likely binding site model for Spodoptera species, based both on the in vitro binding and the in vivo competition among Vip3 proteins.

## Materials and methods

### Expression of Vip3 proteins and engineering of DIP mutants

All Vip3 proteins contained an Nt 6x-His tag. Vip3Aa16 (ac. no. AAW65132) was expressed in recombinant *Escherichia coli* BL21 cells^34^ following the protocol described elsewhere^35^. Vip3Ca2 (ac. no. AEE98106)^22^, Vip3Af1 (ac. no. CAI43275) and mutant Vip3Af E168A (kindly provided by BASF Belgium Coordination Center – Innovation Center Ghent) were expressed in recombinant *E. coli* WK6 cells following a protocol described previously^36^.

For constructing the Vip3 disabled proteins, the mutation S164C L166C was selected based on the results reported by Jerga et al*.*^21^ where a Vip3Aa DIP variant was generated by the introduction of two cysteines in enough proximity to make a S–S bond and prevent Domain I to unfold, a critical step in the activation of the Vip3 protoxins^11,12^. The mutation E168A was selected based on previous studies showing that Vip3Af E168A loses toxicity but retains the tetrameric structure and we hypothesized that could also behave as a DIP mutant^14,16^. The DIP proteins Vip3Aa S164C L166C, Vip3Aa E168A and Vip3Ca S164C L166C were generated for this work by SDM following the protocol described elsewhere^37^, using the WT clones as templates. Primers and annealing temperatures used for SDM Polymerase Chain Reaction (PCR) are collected in Supplementary Table S2. The resulting mutant clones were expressed and lysed following the same conditions used for their WT homologues.

### Purification of Vip3 proteins

For the in vitro binding assays, proteins were purified by metal-chelate affinity chromatography using His-trap FF crude (GE Healthcare) 1 ml columns^35^. Briefly, the clarified lysate was loaded onto the column previously equilibrated with 50 mM phosphate buffer containing 300 mM NaCl and 10 mM imidazole. Then, the column was washed with the same buffer and an increased imidazole concentration (45 mM), and the His-tagged Vip3 protein bound to the column was finally eluted rising imidazole concentration to 250 mM in the same buffer. Purified proteins were dialyzed overnight against 20 mM Tris–HCl, 150 mM NaCl (pH 8.6) buffer, and after dialysis, they were clarified by centrifugation (16,100×*g*, 4 °C, 10 min), quantified by the Bradford method^38^ using bovine serum albumin (BSA) as standard, aliquoted and stored at − 20 °C. The purity of the protein preparations was checked by SDS-PAGE (Supplementary Fig. S2). Just before use, purified Vip3 proteins used as competitors were trypsin-treated (1% w/w) with trypsin from bovine pancreas (SIGMA T8003, Sigma-Aldrich, St. Louis, MO, USA) at 37 °C for 1 h, clarified by centrifugation (16,100×*g* for 10 min at 4 °C), and quantified by Bradford.

In the in vivo competition assays, we observed that His-trap purified proteins rendered less reproducible results compared to the isoelectric point precipitation (IPP) purified proteins, most probably due to a higher stability of the latter on the surface of the diet for 10 days at 25 °C. For this reason, proteins meant to be used for in vivo competition assays were purified by IPP^35^. Briefly, the clarified lysate was lowered at pH 5.6 for Vip3Aa and Vip3Af (WT and mutant proteins) and at pH 5.9 for Vip3Ca (WT and mutant protein) with 0.1 M acetic acid. Then, the lysate with the adjusted pH was aliquoted and the precipitation pellet was recovered by centrifugation (16,100×*g*, 4 °C, 10 min) and stored at – 20 °C. Before being used in bioassays, the pellet aliquots were dissolved in 20 mM Tris–HCl, 150 mM NaCl (pH 8.6) buffer, and clarified by centrifugation (16,100×*g*, 4 °C, 10 min). Protein purity was checked by SDS-PAGE (Supplementary Fig. S3) and the protein concentration was calculated densitometrically using BSA as standard and the TotalLab 1D v 13.01 software.

### *S. littoralis* brush border membrane vesicles (BBMV) preparation

Last instar larvae of *S. littoralis* were dissected and midguts were kept at − 80 °C. BBMV were prepared from the midguts by the differential magnesium precipitation method^39^, flash frozen in liquid nitrogen, and stored at − 80 °C until use. The protein concentration in the BBMV preparations was determined by Bradford.

### Vip3Aa ^125^I radiolabeling

Radiolabeling of Vip3Aa protoxin was performed by the chloramine T method^40^ following the protocol described by Chakroun and Ferré^7^ with some modifications. His-trap purified Vip3Aa (25 µg) was mixed with 0.3 mCi of [^125^I]-NaI and 1/3 (vol/vol) 18 mM Chloramine T. The excess of free ^125^I was separated from the labeled protein using a PD10 desalting column (GE Healthcare). The purity of the ^125^I labelled protein was checked by analyzing the eluted fractions by SDS-PAGE with further exposure of the dry gel to an X-ray film (Supplementary Fig. S4). The specific activity of the labeled ^125^I-Vip3Aa was 5.7 µCi/µg.

### In vitro binding assays with ^125^I -Vip3Aa and ***S. littoralis*** BBMV

Before use, labelled ^125^I-Vip3Aa protein was trypsin treated (1% w/w, 37 °C, 1 h) and stored at 4 °C. BBMV were thawed on ice, centrifuged (10 min at 16,000×*g* and 4 °C) and resuspended in binding buffer (20 mM Tris–HCl, 1 mM MnCl_2_, 0.1% BSA, pH 7.4).

For all the assays, trypsin-treated ^125^I-Vip3Aa was incubated with BBMV for 1 h at room temperature in a 0.1 ml final volume of binding buffer. The reaction was stopped by centrifuging the tubes at 16,000×*g* for 10 min at 4 °C, and the pellet was washed once with 500 µl of cold binding buffer^7^. The radioactivity retained in the pellet was measured in a model 2480 WIZARD2 gamma counter. A binding assay with a fixed amount of ^125^I-Vip3Aa (0.2 nM), increasing concentrations of BBMV and an excess of unlabelled competitor (1000-fold His-trap purified, trypsin-treated, Vip3Aa) was performed to determine the appropriate amount of BBMV to use in the binding competition assays and estimate nonspecific binding (Supplementary Fig. S1). Competition assays were performed by mixing a fixed amount of ^125^I-Vip3Aa (0.2 nM) with increasing concentrations of unlabelled competitor (His-trap purified, trypsin-treated, Vip3 proteins) and a fixed amount of BBMV (0.05 mg/ml). At least three replicates were performed for each binding or competition assay. Graphical representations were performed with GraphPad Prism version 5. Equilibrium dissociation constants (*K*_*d*_) and the concentration of binding sites (*R*_*t*_) were estimated using the LIGAND software^41^.

### Insect rearing and bioassays

Surface contamination bioassays were performed with a laboratory population of *S. littoralis* maintained on semi-synthetic diet^42^ in a rearing chamber at 25 ± 2 °C, 70 ± 5% RH and 16:8 h L:D.

To calculate the LC_50_ values of WT proteins, increasing doses of proteins purified by isoelectric point precipitation (IPP) dissolved in Tris buffer (20 mM, 150 mM NaCl, pH 8.6) were prepared. Protein preparations were dispensed (50 µl) over the surface of the assay wells (2 cm^2^ of diameter) filled with semi-synthetic diet, sixteen wells were used for each protein dose. After the diet surface was dry, one neonate was gently placed into each well and the plates were sealed. Trays were maintained in a climatic chamber at the same conditions used for colony rearing and mortality was recorded after 7 and 10 days of incubation. At least three replicates were performed for each protein, and the LC_50_ values and FL95% were calculated using PoloPlus software.

For in vivo competition assays, protein:competitor ratios of 1:0, 1:10, 1:100 and 1:1000 were prepared in Tris buffer (20 mM, 150 mM NaCl, pH 8.6). Controls consisted of just buffer (labeled as 0) and just competitor (labeled as 0:1000, corresponding to the maximum amount of competitor used in the assay). Constant concentrations of IPP-purified Vip3Aa (13 ng/cm^2^) and Vip3Af (30 ng/cm^2^), which produced 60–80% of mortality, were mixed with the corresponding amount of IPP-purified competitor to prepare the different ratios. Bioassays were performed following the same protocol described previously, and at least three biological replicates were performed for each in vivo competition protein combination. Graphical representations were performed with GraphPad Prism version 5, and for assessing the statistical significance of mortality differences between 1:0 and the rest of protein:competitor ratios, a One way Anova with post-hoc Bonferroni test was performed using the same software.

## Supplementary Information

Supplementary Information.
