# Supplementary material for: In vivo competition assays between Vip3 proteins confirm the occurrence of shared binding sites in Spodoptera littoralis
Source: Sci Rep. 2022 Mar 17;12:4578. doi: 10.1038/s41598-022-08633-y (PMC8931066; doi:10.1038/s41598-022-08633-y)
Supplement: Supplementary file 1 — Supplementary Information. [file 41598_2022_8633_MOESM1_ESM.docx]

***In vivo* competition assays between Vip3 proteins confirm the occurrence of shared binding sites in *Spodoptera littoralis***

María Lázaro-Berenguer, Yudong Quan, Patricia Hernández-Martínez and Juan Ferré^*^

Institute of Biotechnology and Biomedicine (BIOTECMED), Department of Genetics,

Universitat de València, 46100 Burjassot, Spain.

^*^corresponding address: [juan.ferre@uv.es](mailto:juan.ferre@uv.es)

**Running title:** *In vivo* competition among Vip3 proteins

**SUPPLEMENTARY MATERIAL**

**Supplementary Table S1. Binding parameters of ^125^I-Vip3Aa *in vitro* competition binding assays.** The equilibrium dissociation constant (*K_d_*) and the concentration of binding sites (*R_t_*) were calculated for the homologous and heterologous competitors. At least three replicates were used for the data analysis, and the mean values ± SEM are represented.

| **Protein** | ***K_d_* ±SEM (nM)** | ***R_t_* ± SEM (pmol/mg)** |
| --- | --- | --- |
| Vip3Aa WT | 51.0 ± 8.3 | 170 ± 32 |
| Vip3Aa DIP1 | 81 ± 25 | 324 ± 128 |
| Vip3Aa DIP2 | 57.7 ± 11.8 | 190 ± 42 |
| Vip3Af WT | 130 ± 39 | 492 ± 202 |
| Vip3Af DIP | 180 ± 63 | 605 ± 317 |
| Vip3Ca WT | 6.6 ± 1.3 | 18.3 ± 3.4 |

**Supplementary Table S2. List of SDM primers for Vip3 DIP variants generation.** Forward (F) and reverse (R) primers flaking the target sequences and carrying the mutations were designed for introducing the desired modifications. Changed bases compared to the wild type sequence are underlined. The annealing temperature used for each primer pair (Tm) is indicated in °C.

| **Mutant** |  | **Primer sequence** | **Tm** |
| --- | --- | --- | --- |
| **Vip3Aa DIP1 (S164C L166C)** | F | 5’-ATT AAC TGT ACA TGT ACT GAA ATT ACA CCT GCG TAT CAA AG-3’ | 56 |
|  | R | 5’-GTA ATT TCA GTA CAT GTA CAG TTA ATA AGT ACA TTT ACA TTA ATA ATA TCC-3’ |  |
| **Vip3Aa DIP2 (E168A)** | F | 5’-CTT ACT GCA ATT ACA CCT GCG TAT CAA AG-3’ | 56 |
|  | R | 5’-GGT GTA ATT GCA GTA AGT GTA GAG TTA ATA AGT AC-3’ |  |
| **Vip3Ca DIP (S164C L166C)** | F | 5’-ATT AAT TGT ACT TGT ACT GAA ATC ACA CCT TCA TAT CAA CGT-3’ | 56 |
|  | R | 5’-TGT GAT TTC AGT ACA AGT ACA ATT AAT TAA AAC ATT TAA ATT AAC ATC-3’ |  |

**Supplementary Figure S1. Binding assays with ^125^I-Vip3Aa and *S. littoralis* BBMV.** *In vitro* binding assays using increasing concentrations of *S. littoralis* BBMV. Squares and triangles represent total and nonspecific binding (determined with 1000-fold unlabeled Vip3Aa), respectively. Each data point represents the mean of at least three replicates (±SEM).


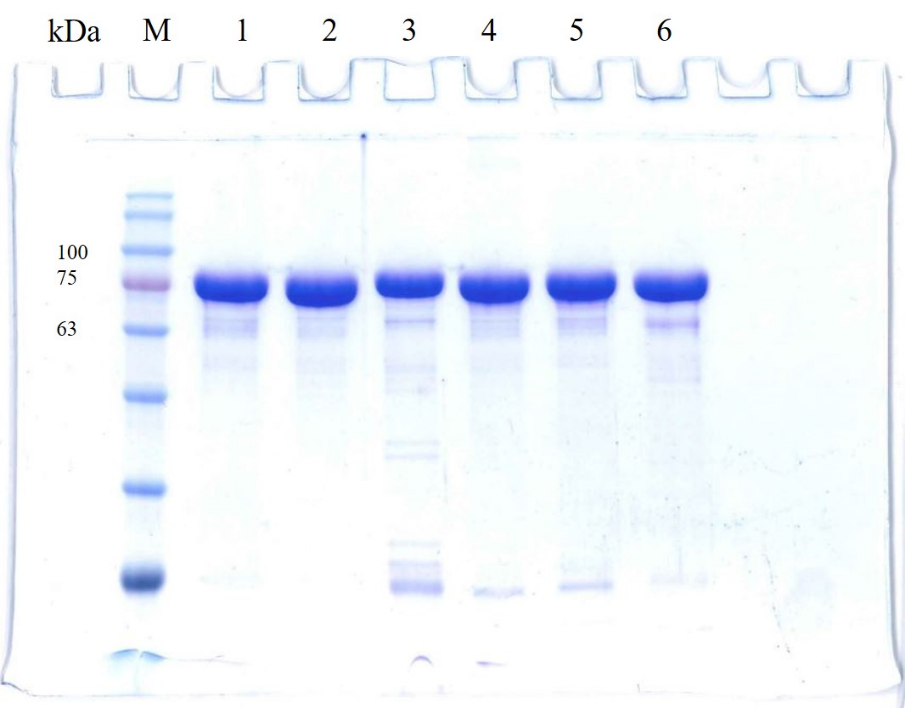


**Supplementary Figure S2. SDS-PAGE of His-trap purified proteins used as competitors for *in vitro* binding assays.** Lanes 1, Vip3Aa WT; 2, Vip3Af WT; 3, Vip3Ca WT; 4, Vip3Aa DIP1; 5, Vip3Aa DIP2, and 6, Vip3Af DIP. M, Blue Star molecular marker.


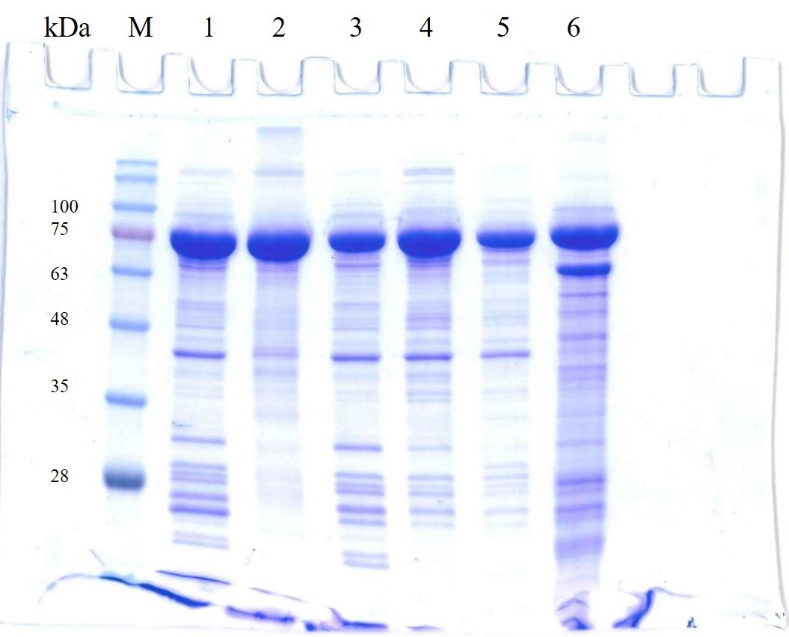


**Supplementary Figure S3. SDS-PAGE of IPP purified proteins used for *in vivo* competitions assays.** Lane 1, Vip3Aa WT; 2, Vip3Af WT; 3, Vip3Aa DIP1; 4, Vip3Aa DIP2; 5, Vip3Af DIP, and 6, Vip3Ca DIP. M, Blue Star molecular marker.


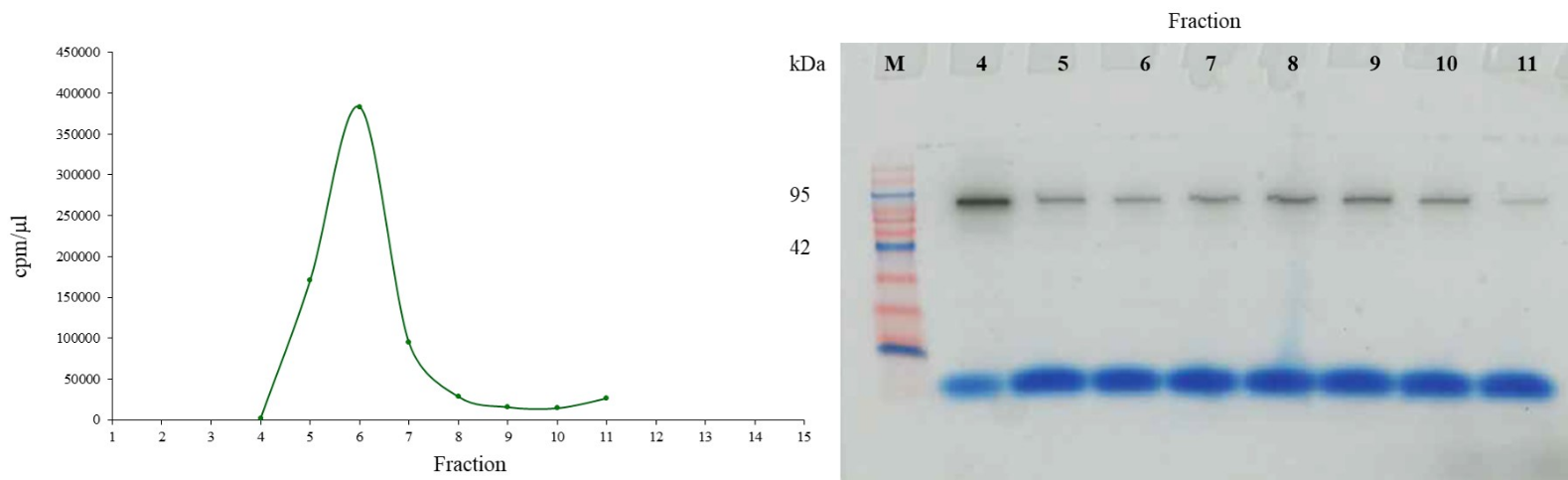


**Supplementary Figure S4. Vip3Aa16 labelling with ^125^I-Na.** A) Separation of the labelled protein by gel filtration on a PD10 column; B) an equivalent number of cpm of each fraction were analysed in SDS-PAGE and autoradiography of the gel was performed.
